# Impact of Timing of Immunotherapy and Cytoreductive Nephrectomy in Metastatic Renal Cell Carcinoma: Real-World Data on Survival Outcomes from the CKCis Database

**DOI:** 10.3390/curroncol31080351

**Published:** 2024-08-18

**Authors:** Changsu Lawrence Park, Feras Ayman Moria, Sunita Ghosh, Lori Wood, Georg A. Bjarnason, Bimal Bhindi, Daniel Yick Chin Heng, Vincent Castonguay, Frederic Pouliot, Christian K. Kollmannsberger, Dominick Bosse, Naveen S. Basappa, Antonio Finelli, Nazanin Fallah-rad, Rodney H. Breau, Aly-Khan A. Lalani, Simon Tanguay, Jeffrey Graham, Ramy R. Saleh

**Affiliations:** 1 McGill University Health Centre, McGill University, Montreal, QC H4A 3J1, Canada; changsu.park@mail.mcgill.ca (C.L.P.);; 2 Cross Cancer Institute, University of Alberta, Edmonton, AB T6G 1Z2, Canada; 3 Queen Elizabeth II Health Sciences Centre, Dalhousie University, Halifax, NS B3H 3A7, Canada; 4 Sunnybrook Odette Cancer Centre, Toronto, ON M4N 3M5, Canada; 5 Southern Alberta Institute of Urology, Calgary, AB T2V 1P9, Canada; 6 Tom Baker Cancer Centre, University of Calgary, Calgary, AB T2N 4N2, Canada; 7 Hotel Dieu de Quebec, Quebec, QC G1R 2J6, Canada; 8 Department of Urology, Centre Hospitalier Universitaire de Québec, Université Laval, Québec, QC G1V 0A6, Canada; 9 Department of Surgery, Université Laval, Québec, QC G1V 0A6, Canada; 10 British Columbia Cancer Agency, Vancouver, BC V5Z 3X7, Canada; 11 Division of Oncology, University of Ottawa, Ottawa, ON K1N 6N5, Canada; 12 Princess Margaret Cancer Centre UHN, Toronto, ON M4W 1H7, Canada; 13 Juravinski Cancer Centre, McMaster University, Hamilton, ON L8S 4L8, Canada; lalania@hhsc.ca; 14 Department of Oncology and Hematology, University of Manitoba, Winnipeg, MB R3T 2N2, Canada

**Keywords:** cytoreductive nephrectomy, renal cell carcinoma, immunotherapy, CKCis, real-world data

## Abstract

Immunotherapy-based systemic treatment (ST) is the standard of care for most patients diagnosed with metastatic renal cell carcinoma (mRCC). Cytoreductive nephrectomy (CN) has historically shown benefit for select patients with mRCC, but its role and timing are not well understood in the era of immunotherapy. The primary objective of this study is to assess outcomes in patients who received ST only, CN followed by ST (CN-ST), and ST followed by CN (ST-CN). The Canadian Kidney Cancer information system (CKCis) database was queried to identify patients with de novo mRCC who received immunotherapy-based ST between January 2014 and June 2023. These patients were classified into three categories as described above. Cox proportional hazards models were used to assess the impact of the timing of ST and CN on overall survival (OS) and progression-free survival (PFS), after adjusting for the International Metastatic RCC Database Consortium (IMDC) risk group, age, and comorbidities. Best overall response and complications of ST and CN for these cohorts were collected. A total of 588 patients were included in this study: 331 patients received ST only, 215 patients received CN-ST, and 42 patients received ST-CN. Patient and disease characteristics including age, gender, performance status, IMDC risk category, comorbidity, histology, type of ST, and metastatic sites are reported. OS analysis favored patients who received ST-CN (hazard ratio [HR] 0.30, 95% confidence interval [CI] 0.13–0.68) and CN-ST (HR 0.68, CI 0.47–0.97) over patients who received ST only. PFS analysis showed a similar trend for ST-CN (HR 0.45, CI 0.26–0.77) and CN-ST (HR 0.9, CI 0.68–1.17). This study examined baseline features and outcomes associated with the use and timing of CN and ST using real-world data via a large Canadian real-world cohort. Patients selected to receive CN after ST demonstrated improved outcomes. There were no appreciable differences in perioperative complications across groups. Limitations include the small number of patients in the ST-CN group and residual confounding and selection biases that may influence the outcomes in patients undergoing CN.

## 1. Introduction

Renal cell carcinoma is the sixth most diagnosed cancer in men and the ninth most diagnosed cancer in women. Its incidence continues to rise approximately 1.5% per year in the United States with an estimated 81,000 expected new diagnoses and 14,000 expected deaths attributable to this disease in 2024 [1]. In the advanced setting, treatment for patients with renal cell carcinoma often requires a multidisciplinary approach with both systemic and local therapies employed to impact the natural history of this morbid disease. 

The treatment paradigm for metastatic renal cell carcinoma (mRCC) has evolved over the last three decades with an increase in median overall survival from approximately 8 months with interferon-based immunotherapy in 1999, to approximately 25 months with targeted therapy in 2007, to approximately 50 months with checkpoint inhibition-based immunotherapy in 2024 [2,3,4,5]. Cytoreductive nephrectomy (CN) has historically been a standard of care, increasing survival by 3–10 months for patients with mRCC receiving interferon-based immunotherapy [6,7]. However, in the era of targeted therapy with tyrosine kinase inhibitors (TKIs), the CARMENA trial demonstrated that sunitinib alone was non-inferior to CN followed by sunitinib in the intention to treat population [8]. Only a small subgroup of intermediate-risk patients benefited from upfront CN in a post-hoc analysis [9]. Furthermore, the SURTIME trial demonstrated that initiation of sunitinib prior to CN was associated with an increased rate of sunitinib treatment and overall survival compared to upfront CN followed by sunitinib, but this trial did not have a comparator arm without CN [10]. Today, the standard of care systemic treatment for mRCC is based on an immunotherapy backbone with a combination of checkpoint inhibition and targeted therapy (Io/TKI) or dual checkpoint inhibition (Io/Io) [11,12,13,14,15]. The role and timing of CN in the era of novel immunotherapy-based systemic treatment (ST) remain controversial. 

Limited observational data show that metastatic patients receiving CN have better survival compared to those who did not receive CN [16,17,18,19]. However, the effect on patient outcomes, CN complications, and ST complications based on the permutations of CN to ST is poorly understood. Using real-world data from the Canadian Kidney Cancer information system (CKCis), we describe outcomes and complications in patients who received ST only, CN followed by ST (CN-ST), and ST followed by CN (ST-CN) in the era of checkpoint inhibition immunotherapy. 

## 2. Materials and Methods

### 2.1. Data Source and Ethics

The Canadian Kidney Cancer information system (CKCis) is a prospectively maintained national database of kidney cancer patients treated at 14 academic centers and 1 community center from 6 provinces. CKCis data have been shown to be representative of the Canadian kidney cancer population and consistent with the American surveillance, epidemiology, and end result (SEER) database [20]. Research ethics board (REB) approval was obtained from each participating center. The lead site for this project was McGill University Health Centre (MUHC); the MUHC REB approval code for CKCis is #10–178-BMA. The study is presented based on the strengthening of the reporting of observational studies in epidemiology (STROBE) statement [21]. 

### 2.2. Patient Population

We identified patients with de novo synchronous mRCC between January 2014 and June 2023 who received front-line systemic therapy with Io/Io (i.e., ipilimumab/nivolumab) or Io/TKI (i.e., pembrolizumab/axitinib; other combinations were not used per regulatory approval in the indexed time period). Synchronous mRCC was defined as histologically proven RCC with radiographical evidence of distant metastasis discovered within three months of diagnosis or curative-intent nephrectomy. All International Metastatic RCC Database Consortium (IMDC) risk categories were included in this study. Patients were categorized into three cohorts: (1) ST only—patients who received immunotherapy-based systemic treatment without CN. (2) CN-ST—patients who started immunotherapy-based systemic treatment for metastatic disease within three months following nephrectomy. Metastatic disease may have been present at the time of nephrectomy or discovered within three months of nephrectomy. (3) ST-CN—patients who received at least one dose of immunotherapy-based systemic treatment prior to nephrectomy for metastatic disease.

### 2.3. Definition of Endpoints and Variables

Overall survival (OS) was defined as the time from initiation of systemic therapy until death from any cause. Progression-free survival (PFS) was defined as the time from initiation of system therapy until disease progression as determined by the treating oncologist or death from any cause. The IMDC score was calculated by retrieving the time to initiation of treatment, Karnofsky performance status, hemoglobin, corrected calcium, neutrophils, and platelets. Each patient was assigned an IMDC risk category of favorable (0 points), intermediate (1–2 points), and poor (≥3 points) as previously described [22]. Best overall response rate per patient was categorized into complete response (CR), partial response (PR), stable disease (SD), and progression of disease (PD) per the Response Evaluation Criteria in Solid Tumors version 1.1 (RECIST v1.1) [23]. The objective response rate (ORR) was defined as the sum of CR and PR. Disease control rate (DCR) was defined as the sum of CR, PR, and SD. The reason for treatment change or cessation was collected. Perioperative complications were defined as medical complications that were directly attributable to the surgical procedure as determined by the treating physicians.

### 2.4. Statistical Analysis

Continuous variables were described using the median and interquartile range. Categorical variables were described using absolute counts and percentages. The OS and PFS distributions were summarized using Kaplan–Meier curves. Median OS and PFS and the corresponding 95% confidence intervals were reported. Log-rank tests were used to compare the survival curves. Cox’s proportional hazards models were used to determine the association of ST and CN on OS and PFS, after adjusting for IMDC risk, age, and comorbidities. The hazard ratio (HR) and the corresponding 95% confidence intervals were reported. A *p*-value < 0.05 was used for statistical significance. Categorical variables were compared using the chi-squared test and ANOVA was used to compare the means between the three groups. Non-normally distributed continuous variables were compared using the Kruksal–Wallis test. All statistical tests were conducted using SAS (SAS Institute Inc., Cary, NC, USA) version 9.4 software.

## 3. Results

### 3.1. Patient Characteristics

A total of 588 patients with mRCC whose data are listed in the CKCis database met the inclusion criteria for this study: 331 (56%) patients received ST only, 215 (37%) patients received CN-ST, and 42 (7%) patients received ST-CN. Patient and disease characteristics for each cohort are listed in Table 1. Patients who were selected to undergo CN regardless of the timing of ST were younger with a median age of 64 in the ST-only cohort versus 62 and 60, respectively, for the CN-ST and ST-CN cohorts (*p* = 0.0001). 

Patients who underwent CN at any time were more likely to have Karnofsky performance status greater than 70 (92% vs. 76%, *p* < 0.0001). The IMDC risk category distribution was similar between the ST-only and ST-CN cohorts. The CN-ST cohort had a more favorable IMDC score compared to the other two cohorts who received upfront ST with 10% vs. 2% in the favorable risk, 64% vs. 42% in the intermediate risk, and 25% vs. 56% in the poor risk category (*p* < 0.0001). The Charlson comorbidity index was similar across all cohorts. Systemic therapy with pembrolizumab and axitinib in the ST only, CN-ST and ST-CN cohorts were 63% vs. 75% vs. 85%, respectively (*p* = 0.0003). The remainder of patients in all cohorts received ipilimumab and nivolumab. The median time to treatment initiation was 1.9 months in the ST-only cohort, 4.9 months in the CN-ST cohort, and 1.5 months in the ST-CN cohort (*p* = 0.044). The rate of metastasis to the brain, lung, and liver were statistically similar in all cohorts. The rate of bone metastasis was higher in the ST-only cohort (42%) compared to CN-ST (29%) and ST-CN (31%) cohorts (*p* = 0.007). The rate of sarcomatoid differentiation in nephrectomy specimens was 30% and 34% in the CN-ST and ST-CN cohorts, respectively.

### 3.2. Survival Outcomes and Response to Treatment

**Figure 1:**
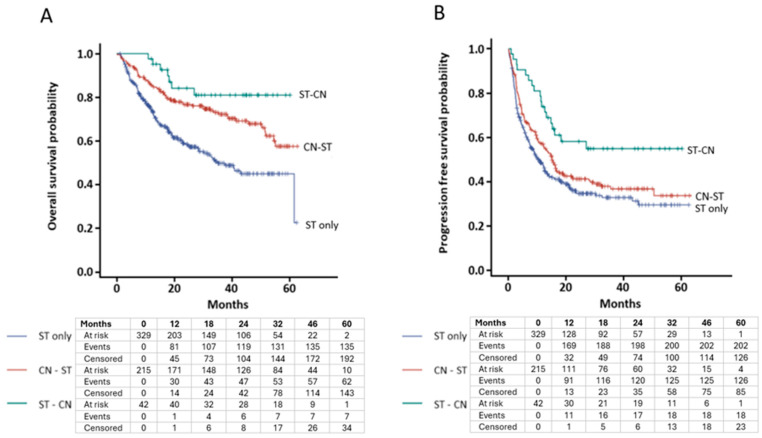
Kaplan–Meier curve for unweighted analysis of survival based on timing of cytoreductive nephrectomy. (**A**) Overall survival (log-rank *p* < 0.0001). (**B**) Progression-free survival (log-rank *p* = 0.004). Below the curve is the number of patients at risk at various landmark time points.

Unweighted analyses of OS (Figure 1A) and PFS (Figure 1B) are presented via Kaplan–Meier curves. Results of multivariable Cox proportional hazard ratios for OS and PFS are listed in Table 2. Multivariable analysis for OS was favored in patients who received ST-CN (HR 0.30, CI 0.13–0.68, *p* = 0.004) and CN-ST (HR 0.68, CI 0.47–0.97, *p* = 0.03) over patients who received ST only. Multivariable analysis for PFS showed a similar association for ST-CN vs. ST only (HR 0.45, CI 0.26–0.77, *p* = 0.004) but not for CN-ST (HR 0.9, CI 0.68–1.17, *p* = 0.44) vs. ST only. Favorable and intermediate IMDC risk category was an independent positive predictor of OS with HR 0.32 (CI 0.13–0.80, *p* = 0.017) and HR 0.47 (CI 0.34–0.65, *p* < 0.0001), respectively, compared to the IMDC poor risk category. The summary of best overall tumor response to systemic treatment in each cohort is reported in Table 3. The objective response rate (ORR) was 32% in ST only, 41.4% in CN-ST, and 59.6% in ST-CN cohorts. The disease control rate (DCR) was 52.8% in ST only, 58.1% in CN-ST, and 71.9% in ST-CN cohorts. Perioperative complications were reported in 45 patients (21%) in the CN-ST cohort and in 6 patients (14%) in the ST-CN cohort (Table 4). There were no unique safety signals in patients who received ST prior to CN.

## 4. Discussion

This study aimed to characterize the real-world practice patterns and outcomes for patients receiving CN as part of their treatment for mRCC in the era of immunotherapy via checkpoint inhibition. While its use is controversial post CARMENA and SURTIME trials, CN remains prevalent with the rate of prior nephrectomy in contemporary trials, being 70% in CHECKMATE-9ER, 83% in KEYNOTE-426, 75% in CLEAR, 80% in JAVELIN renal-101, and 81% in CHECKMATE-214 [11,12,13,14,15]. The proportion of nephrectomies that were performed in the setting of initially localized disease versus true CN in the metastatic setting is not reported. In our study, the rate of CN in the metastatic setting before or after ST was only 43%. This may reflect the changing real-world practice patterns, which are consistent with the lack of level 1 data supporting its use with modern treatments.

In the present study, patients who were selected to undergo CN had improved outcomes compared to patients receiving ST alone. Furthermore, patients who underwent ST-CN had better outcomes compared to patients who had CN-ST, despite negative skewing from the higher proportion of IMDC poor risk category in the ST-CN cohort (55% vs. 25%). The IMDC risk category appears to be prognostically valid in patients receiving contemporary treatments and was an independent predictor of outcome in our multivariable analysis [24]. The ST-CN cohort had the highest proportion of Io/TKI to Io/Io, although the clinical significance of this is unknown as no head-to-head comparisons have been performed for these regimens in mRCC. The ST-CN cohort had the highest overall tumor response to systemic therapy. However, it is unknown whether this response was achieved before or after CN. Excellent response to therapy at metastatic sites may have prompted more nephrectomies to consolidate treatment, which may have led to the selection bias that reflects the improved outcomes in this cohort. Alternatively, removal of the cancer stem cells and immunosuppressive TME via extirpation of the primary tumor may have conferred the survival benefit in these cohorts.

The findings presented in this study should be interpreted within the limitations of the inherent biases of the observational study design that are not fully controlled by our statistical adjustments. Selection bias leading to unequal selection probability into the three cohorts and variables weighing into this decision is more complex than what is reconciled by our multivariate adjustment. This may contribute to the superior ORR in the ST-CN group and the consequent advantages in OS and PFS. However, the PFS in the first three months of systemic therapy was similar across all cohorts, which indicates that initial response was not the major driver in the clinical decision making for patients who received ST-CN. Immortal time bias for patients receiving ST-CN needs to be considered in the OS analysis as patients must be alive and fit to undergo CN post initiation of ST. By definition, patients who died shortly after initiation of ST could not be included in this cohort irrespective of patient or disease characteristics. The limited sample size of the ST-CN cohort and the consequent susceptibility to random error are limitations of this study. As such, more data is required prior to routine implementation of CN in mRCC.

Ongoing randomized control trials will help address some of the biases and limitations of our study. The PROBE trial (NCT04510597) randomizes patients with de novo mRCC to receive immunotherapy-based ST only versus CN within 42 days of initiation of ST. The investigators are assessing OS, PFS, and ORR as their outcome measures. The NORDIC-SUN trial (NCT03977571) includes all patients with de novo mRCC receiving immunotherapy-based ST. At the 3-month and 6-month time intervals, a multi-disciplinary team will determine suitability for CN for patients whose IMDC risk category is ≤3. Selected patients who are eligible for CN will be randomized to continue ST only vs. CN at the designated time intervals. The primary outcome for the NORDIC-SUN is OS. Secondary and exploratory outcomes include various clinical and scientific measures. These two randomized trials aim to answer whether deferred CN is beneficial to patients with mRCC versus ST alone. However, the results of these trials cannot be extrapolated to patients who receive upfront CN and these randomized trials are also not free of the biases that may lead to locoregional therapies for minimal disease burden and ST changes post CN. 

## 5. Conclusions

This study examined baseline features and outcomes associated with the use and timing of CN and ST using real-world data through the CKCis database. Patients who were selected to receive CN after ST had improved outcomes and response to treatment. There were no appreciable differences in perioperative complications based on the timing of ST. Limitations include the small number of patients in the CN-ST group and residual confounding and selection biases that may influence the outcomes in patients undergoing CN. Results of ongoing randomized trials are highly anticipated prior to routinely implementing CN as part of the treatment algorithm for patients with mRCC who are receiving contemporary treatments.

## Figures and Tables

**Table 1:** Patient characteristics.

	ST Only (%)	CN-ST (%)	ST-CN (%)	*p*-Value
Total patients *	331	215	42	
Median age (IQR)	64 (14)	62 (11)	60 (14)	0.0001
Sex				0.135
Male	241 (75)	157 (73)	36 (86)	
Female	90 (25)	58 (27)	6 (14)	
KPS				<0.0001
≥70	235/311 (76)	181/196 (92)	35/39 (90)	
<70	76/311 (24)	15/196 (8)	4/39 (10)	
IMDC				<0.0001
Favorable	6/281 (2)	18/172 (10)	1/33 (3)	
Intermediate	117/281 (41)	111/172 (64)	14/33 (42)	
Poor	158/281 (56)	43/172 (25)	18/33 (55)	
Charlson comorbidity index ‡				0.109
Score 0–2	5/312 (2)	5/202 (2)	1/37 (3)	
Score 3	3/312 (1)	9/202 (4)	0/37 (0)	
Score ≥ 4	304/312 (97)	188/202 (94)	36/37 (97)	
Systemic Treatment				0.0003
Pembrolizumab/axitinib	208 (63)	162 (75)	36 (85)	
Ipilimumab/nivolumab	123 (37)	53 (25)	6 (15)	
Median time to initiation (months)	1.9	4.9	1.5	0.043
Histology				0.019
Clear cell	202/255 (79)	177/201 (88)	29/39 (74)	
Non-clear cell	53/255 (21)	24/201 (12)	10/39 (26)	
Site of metastasis				
Brain	44 (13)	30 (14)	3 (7)	0.482
Lung	242 (73)	161 (75)	27 (64)	0.366
Liver	86 (26)	43 (20)	7 (17)	0.158
Bone	138 (42)	62 (29)	13 (31)	0.007
Median follow-up (months)	17	30	30	

* Total patients reviewed. Each row is normalized to the total available data respectively if missing data for the set variable. ‡ Charlson comorbidity index estimates the 10-year survival in the following scores as 0–2: 90%, 3: 75%, and ≥4: 50% or less. Abbreviations: ST—systemic treatment only. CN-ST—upfront cytoreductive nephrectomy followed by systemic treatment. ST–CN—systemic treatment followed by cytoreductive nephrectomy. IQR—interquartile range. KPS—Karnofsky Performance Status Scale. IMDC—International Metastatic RCC Data Consortium.

**Table 2:** Multivariable cox proportional hazards regression for survival.

	Overall Survival		Progression Free Survival	
	HR (CI)	*p*-Value	HR (CI)	*p*-Value
Age	1 (0.98–1.02)	0.58	0.99 (0.98–1.01)	0.24
IMDC				
Favorable	0.32 (0.13–0.80)	0.017	0.52 (0.28–0.95)	0.03
Intermediate	0.47 (0.34–0.65)	<0.0001	0.86 (0.67–1.1)	0.23
Poor	Reference		Reference	
Charlson comorbidity index				
Score 0–2	0.79 (0.25–2.53)	0.70	0.79 (0.35–1.80)	0.57
Score 3	1.62 (0.51–5.21)	0.42	0.86 (0.35–2.10)	0.73
Score > 4	Reference		Reference	
Treatment				
ST only	Reference		Reference	
CN-ST	0.68 (0.47–0.97)	0.03	0.90 (0.68–1.17)	0.44
ST-CN	0.30 (0.13–0.68)	0.004	0.45 (0.26–0.77)	0.004

**Table 3:** Summary of best overall tumor response to systemic therapy.

Best Response, *n* (%)	ST Only	CN-ST	ST-CN
CR	0 (0)	15 (7.0)	7 (16.7)
PR	106 (32.0)	74 (34.4)	18 (42.9)
SD	69 (20.8)	36 (16.7)	5 (11.9)
PD	88 (26.6)	56 (26.1)	4 (9.5)
Not evaluable/available	68 (20.6)	34 (15.8)	8 (19.0)

CR: complete response; PR: partial response; SD: stable disease; PD: progression of disease. Per Response Evaluation Criteria in Solid Tumors version 1.1.

**Table 4:** Perioperative complications.

	CN-ST *n* = 215 (%)	ST-CN *n* = 42 (%)
Abscess/wound infection	3 (1.5%)	0
Bleeding	5 (2.5%)	1 (2.5%)
Incidental splenectomy	2 (1%)	1 (2.5%)
Deep vein thrombosis	2 (1%)	0
Myocardial infarct	1 (0.5%)	0
Pulmonary embolism	2 (1%)	0
Stroke	1 (0.5%)	0
Pneumothorax	1 (0.5%)	2 (5%)
Dialysis	3 (1.5%)	0
Other *	25 (14%)	2 (5%)
Total	45 (21%)	6 (14%)

* Acute renal injury, adhesions, desmoplastic reaction, difficult dissection, ileus, edema, hematoma, pancreatic leak, pulseless electrical activity arrest, pleural effusion, severe hypotension, vena cava dissection.

## Data Availability

The data presented in this study are available upon request from the corresponding author.
